# Supplementary material for: MicroRNAs are minor constituents of extracellular vesicles that are rarely delivered to target cells
Source: PLoS Genet. 2021 Dec 6;17(12):e1009951. doi: 10.1371/journal.pgen.1009951 (PMC8675925; doi:10.1371/journal.pgen.1009951)
Supplement: Attachment — Submitted filename: Point-to-point response_20210914_for submission.docx [file pgen.1009951.s010.docx]

Following our proposed revision plan and our cover letter to Review Commons dated June 17^th^ 2021, we carefully considered all comments by the four reviewers and modified our manuscript accordingly. In particular, we altered our Abstract to provide a better synopsis of our findings.

A point-to-point response to the specific comments of the four reviewers is included below addressing the comments of the reviewers of Review Commons followed by the forth reviewer of PLOS Genetics.

**Review Commons Refereed Preprint #RC-2021-00655**

We thank the three expert Referees for their appreciation of our work and for their suggestions to improve our manuscript further. Dr. Sara Monaco, Review Commons, assembled the review document^[1]^ and we added our responses here together with experimental revisions. The Referees’ statements, comments and questions are in black, our answers are in blue.

-----------------------------------------------------------------------------

**Reviewer #1 (Evidence, reproducibility and clarity (Required)):**

The current manuscript titled "MicroRNAs are minor constituents of extracellular vesicles that are rarely delivered to target cells" from Albanese et al., show that the majority of extracellular miRNAs from cell culture supernatants are not associated with or contained within EVs, arguing against a role of EVs in transferring functionally relevant amounts of miRNAs to recipient cells to modulate their transcriptome or gene expression profiles. Here, the authors used viral miRNAs released from human B cells latently infected with Epstein-Barr virus as a model to characterize the role of EV-contained viral miRNAs and their known functions in target cells. They also performed spike experiments using cel-miR-39 as an external standard and independent reference to account for variabilities during RNA purification and first-strand cDNA synthesis. They also fused a synthetic, codon-optimized version of the BlaM gene to the carboxy-terminus of CD63 and used for transfection into 293T, Calu-3, Caco-2, HepG2 and Huh7 cells to express CD63-BlaM constitutively. EVs purified from these five different cell lines were incubated with 17 different recipient cells with similar results. Overall this is an interesting manuscript, although mostly describing negative data, which is fine when addressing controversies in the field. My concerns are:

1. If I recall correctly, a majority of miRNA are in the 167K (3hrs) and/or 167K (16hr) preps of ultracentrifugation. Here the authors us the 100K preps (classical exosomes), which make me think that they need to also look into small exosomes or exomeres in the 167K preps for miRNAs.

The focus of our paper is on investigating the presumed role of miRNAs present in extracellular vesicles, i.e., exosomes and microvesicles. For this very reason, our protocol deviates from the classical protocol developed for the preparation of only exosomes as we skipped the 10,000 g step prior to the final sedimentation run at 100,000 g for 2 hours. It was our intention to collect and purify both microvesicles and exosomes (Théry et al., 2006), which is the most standard and accepted way to purify EVs. Publications that state that microRNAs contained in EVs are transferred to and are functional in target cells used an even lower g force or applied a shorter time of centrifugation (Pegtel et al., 2010; Haneklaus et al., 2012; Nanbo et al., 2013). In our manuscript, we show that sedimentation at 100,000 g for 2 hours is sufficient to enrich EVs (or to deplete them from conditioned medium as documented in **Figure 2F**). Of note, EVs were quantitated using the Zetaview instrument (PMX110, Particle Metrix), which cannot properly quantify particles smaller than 60 nm in size (Bachurski et al., 2019). Using our protocol of EV preparation, we analyzed them by electron microscopy and observed vesicles of about 200 nm and particles smaller than 50 nm in size. They represent EVs and particles reminiscent of exomeres, respectively, suggesting that our preparations also contain these sub-EV particles (Zhang and Lyden, 2019), which Reviewer #1 wants us to include in our studies.

2. The ratio of "EV" to "Cell" is not clear throughout the paper. How many EVs per how many cells would give a 50% positive reaction? Again, this is not discussed or planned throughout the paper.

We thank this Reviewer for this comment, which addresses an important issue. In **Figure 5D** we investigated 293T cells and found that 10^4^ VSV-G equipped EVs per cell resulted in 50 % BlaM-positive cells. In our revised manuscript we provide additional information regarding EV concentration and target cell number, which accompany luciferase assays show in **Figure 6A, B and Supplementary Figure S5, S6 and S7.**

3. In plasmid transfections (i.e. ebv-miR-BHRF; Fig 6 and others), it is important to know mRNA copy numbers before making statements on functional effects of miRNA per cell.

We understand the point of this Reviewer and his or her concern. In fact, it is difficult to determine the stoichiometry of miRNA molecules needed to regulate all copies of a given mRNA target. miRNAs tend to bind to and regulate several different mRNA species and, furthermore, a single miRNAs/RISC complex was shown to affect different mRNA molecules consecutively (Hutvágner and Zamore, 2002).

We addressed this question in **Figure 6A** where we show data from reconstruction experiments. The results are derived from luciferase assays using reporter mRNAs and increasing levels of their cognate miRNAs. The graphs in **Figure 6A** show the dose-dependent silencing by two different miRNAs (the amount of transfected, miRNA encoding expression plasmid DNA serves as a proxy for miRNA levels) in combination with two perfect target luciferase reporters, which are expressed at low constant levels. From a single experiment, we also collected total RNA from the transfected cells and performed an absolute quantification of the expressed miRNAs. From this experiment we deduced “*… that, depending on individual reporter plasmids, 20–300 miRNA copies per cell reduced the luciferase activity by half (Supplementary Fig. S8).*” The newly added experimental data shown in **Supplementary Figure S8** is in line with what others have shown previously (Brown et al., 2007).

4. The "Single EV", experiments much like published manuscripts, in Figure 7 are fundamentally flawed, since the cell lines (either donor or recipient) are at different stages of cell cycle (mostly at G1 and some at S/G2/M) with varying secretion properties or uptake. I suggest removing this figure altogether or do a more comprehensive set of experiments on isolating EVs from cells that are blocked using reversible methods (i.e. serum starve for G0 and release for late G1; Hu for G1/S block and release for S; Noco block for G2/M and release for early G1; etc) before making any conclusive conclusions.

Here, we are not sure whether we understand the concerns of Reviewer 1.

In our experiments, cells were counted and if more than 95% of cells were alive we continue with generating EV samples. For this, cells were always seeded at the same initial density (0.5×10^6^ cells/mL) to ensure a reproducible EV production. The non-adherent cells were cultivated for 3 days such that asynchronously proliferating cell populations gave rise to EVs, which were then collected for further analysis. This is the standard, well-established protocol to isolate EVs from cell lines (Pegtel et al., 2010; Nanbo et al., 2013; Meckes et al., 2013). In **Figure 7**, we quantitated the absolute number of released EVs in the supernatants and the absolute number of miRNAs contained in the preparations to calculate the number of individual miRNA species per EV. This experiment was done in a similar fashion by Chevillet et al., for example (Chevillet et al., 2014). In essence, our results are in line with previous publications.

The cell lines investigated here have population doubling times of about 24 to 32 hours with G1 being the dominant phase of the cell cycle. We are unaware of published work that demonstrates a functional link between EV biogenesis and the cell cycle although it is plausible to assume that the release of EVs takes place mostly in G1. It has been reported that single cells secrete about 60 to 65 exosomes per hour on average (Chiu et al., 2016) indicating that EVs accumulate over time in cell culture supernatants. This is why we collected EVs from a production period of 72 hours to integrate EV release from the different phases of the cell cycle.

Reviewer #1 (Significance (Required)):

Overall this is an interesting manuscript, although mostly describing negative data, which is fine when addressing controversies in the field. My concerns are:

1. If I recall correctly, a majority of miRNA are in the 167K (3hrs) and/or 167K (16hr) preps of ultracentrifugation. Here the authors us the 100K preps (classical exosomes), which make me think that they need to also look into small exosomes or exomeres in the 167K preps for miRNAs.


**Reviewer #2 (Evidence, reproducibility and clarity (Required)):**

The advances reported in this study are both conceptual and technical.

**Conceptual:**

- This study, by including numerous negative but also positive controls, is shedding new light EVs fusion efficiency. Indeed, the authors demonstrate that EVs' luminal cargo delivery is highly inefficient in the absence of fusogenic proteins associated with EVs. Although a negative result is difficult to demonstrate per se, the authors are convincingly demonstrating that this EVs fusion event is much less efficient than initially reported.
- The demonstration of functional mRNA, but no miRNA delivery using a similar experimental setup is an interesting advance as these aspects (mRNA vs miRNA) are generally addressed by independent study, thereby making it difficult to compare them. Although the authors would have to more convincingly demonstrate that their miRNA sensor is sensitive enough to detect a few hundred copies of miRNA, this differential function impact of mRNA vs miRNA is interesting per se and is important information for the current effort in the field of EVs bio-engineering, some aiming to deliver mRNA through these circulating carriers.

**Technical:**

- The repurposing of the BlaM assay to detect EVs-fusion events is quite interesting and shows high potential for the field of EVs. This assay was initially developed by Cavrois et al (2014) to monitor HIV-1 viral entry. As mentioned in the manuscript, this assay requires only the engineering (expression of CD63-BlaM) from the EVs producing cells, allowing to test EVs delivery on non-engineered primary cells or cell lines. As nicely demonstrated in Figure 5E, this assay allowed the authors to test an unprecedented combination of donor and acceptor cells, thereby revealing some very interesting cell-specific properties from EVs and their target cells regarding their functional delivery in presence of VSV-G.
- Place the work in the context of the existing literature (provide references, where appropriate).

Numerous publications in the field of EVs are reporting the functional impact of miRNA associated with EVs. However, there are currently very few studies challenging this establishing dogma, most probably because convincingly demonstrating a lack of effect is difficult by itself.

However, it should be noted that, in most cases, studies demonstrating the functional delivery of EVs-associated miRNA are not including some important controls to properly evaluate the direct impact of loaded miRNA, beyond the overall impact of EVs on cells. Indeed, it is clear that EVs themselves can have direct consequences on the biology of cells, notably by triggering signaling events via surface or endosome receptors. EVs-associated RNAs were also reported to trigger innate immunity pathways through TLRs. It is also possible that EVs can have a toxic effect upon their delivery at high doses (also nicely illustrated in this manuscript with EVs from EBV latently infected cells, Fig S7B). Most studies in the literature are not taking enough of this potential effect in their experiment which may lead to triggering non-specific effect on reporters (luciferase, GFP).

We thank Reviewer #2 for bringing up these relevant points. In our manuscript, we confirm that miRNA copy numbers in EVs are low (as shown by others), but we also document that EV fusion events are much less efficient than thought previously. Here, we provide new tools and assays, which are technically easy to follow and which can be used by others in their EV models of choice to investigate whether the observed EV phenotypes are a consequence of delivery of EV-born cargo or are an epiphenomenon. Along the same lines, we also provide and propose several new types of positive and negative controls, which can be adopted by others if needed or appropriate.

The development of new reporter assays for monitoring EV-associated RNA delivery at a single cell level also reported quite inefficient EVs fusion efficiency. For example, de Jong et al (Nature communication, 2020) developed a CRISPR/Cas9 reporter system (CROSS-FIRE) by encapsulating sgRNA in EVs and monitoring activation of a Cas9-dependent fluorescent genetic sensor in Cas9 expressing cells upon EVs delivery. They could only observe 0.06% of cells with activated sensors after 9 days of daily EVs treatment. Of note, in these experiments, they used an average dose of 15 Million EVs per cell for each daily application, which is at least 2 log of magnitude higher than in the present study. As such, it is unlikely that in the present study, the authors could obtain a better efficiency by applying a higher dosage, here not performed because of their toxic impact on cells (**Fig. S7B**).

Of note, Somiya et al (Somiya and Kuroda, 2021) have described a new reporter assay to monitor delivery of EV contained cargo while our manuscript was under consideration. Their experiments are based on a split luciferase approach (HIBIT system). The authors detected EV cargo delivery, only, when EVs were pseudo-typed with VSV-G, which is fully in line with our data supporting our conclusions.

Our new EVs fusion reporter system (CD63-BlaM) together with the previously described CROSS-FIRE and HIBIT systems will allow to better understand EV function(s) and could help to identify proteins, drugs, or physiological cues to improve EVs fusion efficiency.

- State what audience might be interested in and influenced by the reported findings.

This work will be of high interest to investigators in the field of extracellular vesicles and extracellular RNAs. The study also reports important observation in the field of virology, notably by exploring the potential impact of EVs-borne RNA produced from EBV-infected cells.

- Define your field of expertise with a few keywords to help the authors contextualize your point of view. Indicate if there are any parts of the paper that you do not have sufficient expertise to evaluate.

RNA and extracellular RNA biology, EVs biology.

Reviewer #2 (Significance (Required)):

The advances reported in this study are both conceptual and technical.

**Reviewer #3 (Evidence, reproducibility and clarity (Required)):**

The manuscript "MicroRNAs are minor constituents of extracellular vesicles that are rarely delivered to target cells" by Manuel Albanese, Yen-Fu Adam Chen, et al addresses the challenging question of whether there is physiologically meaningful transfer of microRNAs between cells via extracellular vesicle (in particular those in the size and density range of exosomes). While there is much skepticism in the microRNA field about such transfer, papers suggesting as much continue to be published, some in high impact journals. Skepticism is well founded given the microRNAs are thought to act stoichiometrically rather than catalytically and previous work has shown that very few miRNA molecules are loaded into exosomes (see citations in paper). Therefore, it is hard to imagine how enough microRNA could be transferred between cells to have a physiological consequence on the recipient cell. This manuscript provides further evidence that skepticism for microRNA transfer between cells via exosomes is well warranted.
The paper provides three important pieces of such evidence:

1) again showing miniscule amounts of microRNA are loaded into exosomes,

2) that the contents of exosomes are not efficiently directed into the cytoplasm of recipient cells where miRNAs would be expected to act, and

3) even when vesicles are artificially induced to fuse with recipient cells by introducing VSV-G, not enough microRNA is transferred to suppress an highly sensitive reporter in those cells. While the first piece of evidence has been reported, it is nice to have further confirmation. The second two pieces of evidence are more novel and thus should be of greater impact. Overall, the experiments throughout the paper are well done and quite convincing. Importantly they tested transfer between multiple different combinations of donor and recipient cell lines.

**Specific comments:**

1. EM of vesicles in figure 1E is of low quality. Not clear if this issue is due to vesicle prep, EM prep, or the images themselves. Would be nice to have a more convincing image. Following, the iodixanol density gradient, the vesicles should be quite pure.

We think that theses EM images are in line with what has been published by others. As a consequence of iodixanol density gradient centrifugation, we also see particles of the size of exomeres in the range of ≤50 nm together with other types of EVs of around 200 nm similar to (fractioned) preparation in a recent publication (Zhang and Lyden, 2019). Following the suggestions of Reviewer #4 we highlighted EVs in **Figure 1E** using arrowheads in the mixed population of 200 and ≤50 nm particles.

2. Why no Bioanalyzer trace/RT-qPCR for iodixanol density prepped vesicles in figure 2?

In **Figure 2**, we focus on the characterization and quantification of miRNAs in samples prior to and after density gradient centrifugation. This figure documents that the majority of miRNAs do not co-purify with EVs. qPCR quantifications of miRNAs contained in EV fractions 2 and 3 after density gradient centrifugation are shown in **Figure 7** in our manuscript. Panel A shows that miRNA contained in highly purified EVs are very scarce.

We also performed Bioanalyzer runs with RNA extracted from selected fractions after density gradient centrifugation. We did not include the resulting electropherograms in our revised manuscript, but an example is provided below. We can include it (and additional electropherograms) in our revised manuscript if this Reviewer thinks that these plots provide additional valuable information.

Marker

Figure 1: Electropherogram of fraction 2 after iodixanol density gradient centrifugation using a Bioanalyzer instrument. The extinction profile suggests a substantial enrichment of small RNA species in this fraction as expected.

3. Not clear what the different gradient fractions in figure 5C represent. Do not match fraction number in figure 3D and no independent analysis was done. Importantly, the number of fractions with dye and BlaM transfer seems very high. Vesicles should only be in 1 or 2 fractions. Authors should explain.

This is a valuable hint, which we addressed in the revised manuscript to understand the situation in **Figure 5C** better. The explanation is simple but not immediately obvious:

In our iodixanol density gradient preparations most EVs are in fractions 2 and 3 as shown in **Figure 1B**, but some are also present in the subsequent fractions 4 and 5 although at considerably lower levels (**Fig. 1B**). Experiment shown in **Figure 5C** were designed to be able to identify (infrequent) fusion events of unmodified EVs, i.e., those without VSV-G. For this very reason, we used as many EVs as possible (i.e., fixed volume aliquots) from fractions 2 and 3 as well as from the following fractions to target cells. As a consequence, we also detect binding and fusion with aliquots from fractions 4 and 5 in both panels of **Figure 5C**. Fusion events, however, are only apparent when EVs were used that had been equipped with VSV-G. (Yet another consequence of this protocol are very high levels [up to 100 %] of PKH26- and BlaM-positive cells in fractions 2 to 4.)

With this finding, two points appear worth emphasizing: (i) The newly introduced EV fusion assay is very sensitive and allows detection of fusion at extremely low concentrations of VSV-G equipped EVs. (ii) PKH26 staining, which has been used so far to study EV-mediated delivery of cargo is unreliable as it identifies EV binding, only.

4. Figure 6B: The fact that miR-BART1 increases luciferase activity in cells receiving the vesicle delivered miR-BHRF1-2 reporter (and vice-versa for miR-BHRF1-2 with miR-BART1 reporter) is surprising. Can the authors explain?

This assay measures the EV-mediated transfer of luciferase mRNA to recipient cells. Twenty-four hours prior to EV transfer, the recipient cells were transiently transfected with miRNA encoding expression plasmids. Control cells (Ctrl), however, have been transfected with the backbone plasmid such that they do not ectopically express either miR-BART1 or miR-BHRF1-2. In the revised version of our manuscript, we repeated the experiments and used additional five unrelated miRNA encoding expression plasmids to better control the experimental setting and provide comparative (control) data. The results are shown in the revised **panel B of Figure 6**. The new data document that recipient cells expressing the specific miR-BART1 and miR-BHRF1-2 miRNAs reduce luciferase activity in comparison to five unrelated miRNAs (miR-BART2, miR-BART3, miR-BART4, miR-BART22, miR-BHRF1-1).

Reviewer #3 (Significance (Required)):

While it is impossible to rule out that physiologically meaningful transfer of microRNAs does not occur in any context, reporting of these findings is important so that the proverbial bar is appropriately elevated for papers attempting to claim such transfer.

**Reviewer #4 (PLOS Genetics):**

Comments to the Authors:

Reviewer #1: In this manuscript, Albanese M et al. aimed to investigate the capacity of extracellular vesicles (EVs) to deliver packaged miRNAs into target cells and analyze if EV-contained miRNAs could affect the recipient's target mRNA. The authors argue that miRNAs are rarely present in EVs. Unlike several studies, the authors performed a series of experiments, including a new sensitive assay that applied a synthetic, codon-optimized version of the BlaM with CD63 for fusion analysis in the recipient cells, to suggest EV fused with host cells with low efficiency, not to mention to deliver the EV borne miRNAs into recipient cells. Overall, this study provides biochemical evidence to suggest EVs are associated with a low level of extracellular miRNAs. The manuscript is well written and presented. I am intrigued by numerously negative results present in this manuscript to challenge EV-mediated miRNA function to regulate target mRNA in recipient cells. I think the previous three reviewers' comments in the preprint are valid and relevant, and the authors also seem to respond reasonably. Additionally, I suggest a bit more revised experiments and points to address or discuss below:

Major concerns:

1. Most of the recipient cell lines presented in this study are immortal cell lines, which proliferate robustly. This might affect EV fusion and uptake in the recipient cells while under active proliferation. Could the authors try the EBV experiment in more physiological cell settings, such as primary senescent MEFs or postmitotic cells, such as neurons?

We understand the concern of the reviewer. To address it we tested several cell lines of different origins, providing an unprecedented amount of combination of donor to target cells tested (see **Figure 5E**). To study postmitotic cells and primary cells in a more physiological cell context we used Peripheral Blood Mononuclear Cell (PBMCs) freshly isolated from blood of human volunteers (see **Figure 5F**). PBMCs contain different types of cells, which are not proliferating, some of which have been reported to take up EVs such as monocytes or pDCs (Pegtel et al., 2010). Using our novel CD63-BlaM assay, we mostly failed to confirm the previous findings and only a very low percentage of dendritic cells (DCs) and plasmacytoid dendritic cells (pDCs) seem to turn ‘blue’ (**Fig. 5F, left part**). Only when incubated with CD63-BlaM assembled EVs equipped with VSV-G, a substantial transfer of protein cargo could be demonstrated mainly with monocytes, DCs, and pDCs whereas lymphocytes were less efficiently targeted (**Fig. 5F, right part**).

2. Luciferase assay is standard and sensitive for testing miRNA targeting, but the stoichiometry between miRNA and targeting also affects the luciferase activity. In Figure1~3, EVs associate with minimal miRNAs, and, predictably, there is no change in Figure 6C. Is that possible to examine the spike in miRNAs in the recipient cells by RNAseq?

We failed to see any effect using the sensitive luciferase assay using EVs obtained from 293T or primary LCLs (**Figure 6** and **Supplementary Figure 6**). Therefore, we decided to quantify the miRNAs content in these EV preparations as shown in **Figure 7**. In this figure, we used specific stem-loop Taqman probes for the four miRNAs of interest. To our knowledge this technology is the most sensitive and specific way to quantify miRNAs. As this Reviewer suggested, we initially considered to do RNA-seq with our EV preparations because RNA-seq would give a better overview of the general miRNA content in these preparations. However, RNA-seq is not as sensitive as is a stem-loop Taqman approach (unless one invests into an extreme sequencing depth and read coverage). Moreover, the stem-loop Taqman approach provides an absolute quantification of the specific miRNAs of interest. Therefore, we would like to stick to the superior technology as shown in **Figure 7**.

3. While I think the biochemical data presented in this study are convincing, the authors did not do a fair job of discussing their data with limitations. For example, the data are presented in limited cell lines, with all in vitro artificial settings. The design of EBV and virus RNA might not entirely reflect the host cell-secreted EVs. Further, some miRNAs might be tailed or with the addition/editing of the nucleotide to facilitate sorting into exosomes, which is not factored in within this study. The authors might need to discuss the shortage of their setting to balance the views of secretory miRNAs.

Throughout our study we made use of newly established lymphoblastoid cell lines, i.e., primary B cells immortalized by EBV as model to produce and characterize miRNAs released by these cells. In fact, this is a well-accepted model (Baglio et al., 2016; Pegtel et al., 2010), which – as far one knows - resembles as much as possible EVs released by EBV infected cells *in vivo*. The viral miRNAs are expressed from their natural genetic positions in the EBV genome suggesting that their expression is under the control of viral promoters, which are also active *in vivo*. Moreover, these miRNAs have been already reported to be enriched and functional in EVs released by LCLs (Pegtel et al., 2010; Haneklaus et al., 2012), this is the reason why we started characterizing them.

Following this Reviewer’s suggestion, we took them into account and discuss them in the revised manuscript in the Discussion section.

Minor concerns:

1. The manuscript mentions "data not shown" in the points to the reviewer and the discussion. Please show them.

This comment refers to a technically difficult and intricate quantification of miRNAs per cell. Both this Reviewer and Reviewer #1 commented on this issue. We include the result of such an experiment in the revised version of our manuscript. The data are shown in the newly added **Supplementary Figure 8** replacing our "data not shown" statement as requested.

2. In Figure 1D, how is the RNA percentage quantified? It looks to me that the total RNA is not reaching 100%. Additionally, in Figure 1E, EVs in the negative stain data look very heterogeneous. It will be good to annotate them with arrowheads to indicate which are EVs.

In **Figure 1D**, the sum of all fractions for the four miRNAs is 100%. We provide an extra Excel file with the raw data in the context of this rebuttal to document this fact.

Regarding **Figure 1E**, we thank the Reviewer for the suggestion. We added arrows as requested to indicate EVs for readers who are not familiar with the visualization of EVs using negative stained electron microscopic images.

Cited literature

Bachurski D, Schuldner M, Nguyen PH, Malz A, Reiners KS, Grenzi PC, Babatz F, Schauss AC, Hansen HP, Hallek M, Pogge von Strandmann E (2019) Extracellular vesicle measurements with nanoparticle tracking analysis - An accuracy and repeatability comparison between NanoSight NS300 and ZetaView. J Extracell Vesicles 8:1596016. doi:10.1080/20013078.2019.1596016

Baglio SR, van Eijndhoven MA, Koppers-Lalic D, Berenguer J, Lougheed SM, Gibbs S, Léveillé N, Rinkel RN, Hopmans ES, Swaminathan S, Verkuijlen SA, Scheffer GL, van Kuppeveld FJ, de Gruijl TD, Bultink IE, Jordanova ES, Hackenberg M, Piersma SR, Knol JC, Voskuyl AE, Wurdinger T, Jiménez CR, Middeldorp JM, Pegtel DM (2016) Sensing of latent EBV infection through exosomal transfer of 5’pppRNA. Proc Natl Acad Sci U S A 113:E587–96. doi:10.1073/pnas.1518130113

Brown BD, Gentner B, Cantore A, Colleoni S, Amendola M, Zingale A, Baccarini A, Lazzari G, Galli C, Naldini L (2007) Endogenous microRNA can be broadly exploited to regulate transgene expression according to tissue, lineage and differentiation state. Nat Biotechnol 25:1457–1467. doi:10.1038/nbt1372

Chevillet JR, Kang Q, Ruf IK, Briggs HA, Vojtech LN, Hughes SM, Cheng HH, Arroyo JD, Meredith EK, Gallichotte EN, Pogosova-Agadjanyan EL, Morrissey C, Stirewalt DL, Hladik F, Yu EY, Higano CS, Tewari M (2014) Quantitative and stoichiometric analysis of the microRNA content of exosomes. Proc Natl Acad Sci U S A 111:14888–14893. doi:10.1073/pnas.1408301111

Chiu YJ, Cai W, Shih YR, Lian I, Lo YH (2016) A single-sell assay for time lapse studies of exosome secretion and cell behaviors. Small 12:3658–3666. doi:10.1002/smll.201600725

Haneklaus M, Gerlic M, Kurowska-Stolarska M, Rainey A-A, Pich D, McInnes IB, Hammerschmidt W, O’Neill LAJ, Masters SL (2012) Cutting Edge: miR-223 and EBV miR-BART15 regulate the NLRP3 inflammasome and IL-1beta production. J Immunol 189:3795–3799. doi:10.4049/jimmunol.1200312

Hutvágner G, Zamore PD (2002) A microRNA in a multiple-turnover RNAi enzyme complex. Science 297:2056–2060. doi:10.1126/science.1073827

Meckes DG, Gunawardena HP, Dekroon RM, Heaton PR, Edwards RH, Ozgur S, Griffith JD, Damania B, Raab-Traub N (2013) Modulation of B-cell exosome proteins by gamma herpesvirus infection. Proc Natl Acad Sci U S A 110:E2925–33. doi:10.1073/pnas.1303906110

Nanbo A, Kawanishi E, Yoshida R, Yoshiyama H (2013) Exosomes derived from Epstein-Barr virus-infected cells are internalized via caveola-dependent endocytosis and promote phenotypic modulation in target cells. J Virol 87:10334–10347. doi:10.1128/JVI.01310-13

Pegtel DM, Cosmopoulos K, Thorley-Lawson DA, van Eijndhoven MA, Hopmans ES, Lindenberg JL, de Gruijl TD, Wurdinger T, Middeldorp JM (2010) Functional delivery of viral miRNAs via exosomes. Proc Natl Acad Sci U S A 107:6328–6333. doi:10.1073/pnas.0914843107

Somiya M, Kuroda S (2021) Real-time luminescence assay for cytoplasmic cargo delivery of extracellular vesicles. Anal Chem 93:5612–5620. doi:10.1021/acs.analchem.1c00339

Théry C, Amigorena S, Raposo G, Clayton A (2006) Isolation and characterization of exosomes from cell culture supernatants and biological fluids. Curr Protoc Cell Biol Chapter 3:Unit 3.22. doi:10.1002/0471143030.cb0322s30

Zhang H, Lyden D (2019) Asymmetric-flow field-flow fractionation technology for exomere and small extracellular vesicle separation and characterization. Nat Protoc 14:1027–1053. doi:10.1038/s41596-019-0126-x

1. In the original review document assembled by Review Commons, comments by Reviewer #2 seem to be present twice. We removed the duplicated parts in our compilation here.
