# Supplementary material for: MicroRNAs are minor constituents of extracellular vesicles that are rarely delivered to target cells
Source: PLoS Genet. 2021 Dec 6;17(12):e1009951. doi: 10.1371/journal.pgen.1009951 (PMC8675925; doi:10.1371/journal.pgen.1009951)
Supplement: Attachment — Submitted filename: Point-to-point response_20211110_for submission.docx [file pgen.1009951.s011.docx]

Second revision

Point-to-point response

We thank Reviewer 3 for the affirmative evaluation of our work and his/her very thoughtful comments on specific, mostly technical issues. We also thank all Referees of our manuscript for their scientific evaluation and great support.

A point-to-point response is included below addressing the comments of Reviewer 3, which, unfortunately, were not communicated in the first revision of our manuscript. Following the editors’ advice, we fixed our manuscript regarding comments 3, 4, 5, and 6 and addressed comment 1 partially.

The Reviewer’s statements, comments and questions are in black, our answers are in blue.

-----------------------------------------------------------------------------

**Reviewer #3**

**EVIDENCE, REPRODUCIBILITY AND CLARITY**

… … …

During the first round of review, I initially suggested two additional experiments to improve the manuscript.

1/ An interesting observation in this manuscript is the demonstration of functional delivery of mRNA, but not miRNA, when using VSV-G functionalized EVs. As commented by the authors, one hypothesis for the lack of EV-borne miRNA is their low abundance in EVs, as reported in many publications in the field. To support this hypothesis, it would be interesting that the authors determine the copy number / EVs of their encapsulated reporter mRNA. Would a higher copy number per EVs than for miRNA explain the increased functionality? Or can it just be explained by the fact that a much lower mRNA copy number is required for their detection assay (as luciferase assays are very sensitive in this context). The authors already produced a batch of VSV-G EVs containing luciferase reporter mRNA, and also demonstrated their capacity to count EVs and absolute miRNA copy numbers from different conditioned medium fractions. These experiments would involve qRT-PCR and RNA spike controls, and as such are relatively inexpensive.

This is an interesting thought at first glance but, to us, it seems to be a detail, which is distantly connected to our topic. Our manuscript is on miRNAs and their transfer to cells using EVs as vehicles. We report that (i) EVs have no detectable fusogenic capacity. Our manuscript also confirms previous work that (ii) the copy number of miRNAs per EV is very low, and thus likely too low to regulate cellular transcripts in target cells even if one ignores (i) but assumes that EVs were capable of fusing with cells delivering the miRNA cargo.

Our work with EBV (doi: 10.1073/pnas.1115906109) and also work by Tom Shenk’s group (doi: 10.1126/science.288.5475.2373) document that enveloped DNA viruses (which do have fusogenic capacity in contrast to EVs) can also deliver messenger RNAs to target cells. This observation inspired us to (iii) use an mRNA which encodes a traceable reporter (luciferase) and (iv) test its EV-mediated delivery in our model here. Since we found that EVs fail to deliver miRNAs in our experiments (Figures 5 and 6), we needed a positive control to prove this hypothesis in principle. Towards this end we engineered EVs equipped with VSV-G. This idea worked well and the luciferase mRNA is translated upon its delivery as documented in Figure 6.

Reviewer 3 suggests an extension of this control experiment and asks to determine the “copy number / EVs of their encapsulated reporter mRNA”. We are, however, uncertain what one might gain from knowing the answer to this question. The information would be a figure, only, that does not take into account the number of EVs that are taken up by the cells. This number also does not consider the fraction of EVs that contains VSV-G versus the fraction of EVs that does not (the EV producing cells are transiently transfected with a VSV-G encoding expression plasmid). Without this information, this number would not mean much and the suggested experiment would be incomplete.

We have established a ‘nano’ flow cytometry technology to measure fractions of EVs that carry (viral) glycoproteins within a heterogeneous EV preparation (or a virus stock). For this we use carefully validated, proprietary antibodies against, for example, Spike of SARS-CoV-2 or glycoproteins of EBV. We have no good antibodies directed against VSV-G and, from our work with both SARS-CoV-2 and EBV, we doubt that they are commercially available. This is because the surface area of an EV is smaller by a factor of 10,000 on average compared to the surface area of a cell. It is difficult to identify an antibody that provides the necessary sensitivity in nano flow cytometry.

This situation leaves us with two options:

A. We try to work on this point. For this we will have to establish a quantitative RT-qPCR to measure the absolute number of luciferase transcripts per EV in a given preparation. Next, we have to apply nano flow cytometry to determine the fraction of VSV-G positive EVs in this preparation to normalize the data and calculate the absolute number of luciferase transcripts per VSV-G positive EV. Assuming that a VSV-G specific antibody is available that works in nano flow cytometry we might be done with this experiment at the turn of the year at best. If a suitable antibody does not exist or is not available from commercial or academic sources this idea cannot be realized without going through establishing a new (monoclonal) VSV-G specific antibody.

B. Alternatively, we comment on this point in our revised manuscript. Therefore, we introduced the following statement in the Result section to address this issue: *These data demonstrate that the content of EVs is potentially functional when it is successfully delivered to the cytoplasm of recipient cells but such delivery is rare. Based on similar experiments (Jochum et al., 2012), we have estimated that the dose of EVs assembled with VSV-G lead to fewer than one hundred mRNA molecules encoding the luciferase reporter enzyme being delivered to the recipient cells. These estimates are necessarily imperfect; they reflect the uncertainties in the dose, the content of RNA in the EVs, the fraction of the EVs carrying VSV-G, and the rate of uptake of the EVs. Some of these parameters such as the fraction of VSV-G positive EVs or the rate of EV uptake are difficult to assess experimentally.*

2/ I would suggest the authors demonstrate the sensitivity of their miRNA reporter assay by assembling a reporter for low expressed miRNA (200 copies per cell or less, as initially determined by RNA-seq and absolute miRNA count per cell) and evaluate its performance in this context. Alternatively, establishing a stable line expressing the reporter at a low level, or using lentiviral transduction at low MOI and/or a low activity promoter would allow obtaining more physiologic expression level. Establishing a new reporter for low-abundance miRNA and testing it by transient transfection can be achieved in 1-2 weeks, and at low cost (a few oligos, and cloning steps followed by transient transfection). Establishing stable cell lines expressing viral miRNA reporter at low expression level would be longer (1 month) but could lead to a more sensitive cell-based assay for detecting a low-copy number of EV-borne viral miRNA delivered by EVs.

Reviewer 3 ‘comments’ on this point regarding our revised version (Oct 21) by stating that “… the author successfully addressed my point of concern related to the sensitivity of their miRNA reporter assay. In the new figure S8, they could convincingly demonstrate a 50% reduction in luciferase activity with around 20-300 miRNA/cells, and therefore that they can detect the functional impact of low expressed miRNA.“ Therefore, we believe that our first revision has answered this Reviewer’s concern already.

I also had minor comments that could be addressed by the author to improve the manuscript:

3/ In several figures (at least Fig. 1, 3, 4, S6, S7), the authors present a representative experiment among 2 or 3 executed. It would be better to present graphs representing the average value among replicates (including error bars), or at least to include the additional dataset as supplementary data.

Clearly, there is variability between different EV preparations, which is already known to be a problem in the field, especially when dealing with primary cells (see also MISEV2018; https://www.tandfonline.com/doi/full/10.1080/20013078.2018.1535750). Combining results from different EV preparations with their known variability would require normalization with external standards prior to their averaging. As a consequence, we would dismiss the option to show absolute quantitation of miRNAs and EVs studied under different experimental conditions. Therefore, we do not agree with this Reviewer’s suggestion to present graphs representing the average value among replicates, but decided to show one example out of 2/3 replicates. In this second revision, we include a word file that collects and shows different replicates from these experiments. As one can see, the tendency is similar between the different experiments, but the absolute numbers differ. We think, however, that including our additional data with the many biological replicates does not provide much additional information. We therefore would like to suggest to not include them.

4/ Figure 5E, and S4A: The authors mention that this experiment was replicated 3 times. It is however unclear whether it is technical replicates (EVs prepped only once, but transfer assay conducted 3 times in parallel, or whether this is a biological replicate (multiple EVs preparation, and independent test on acceptor cells). Given the importance and interest of this key experiment, it is important to precise as variability in the assay could impact some of the conclusions reported in the heatmap.

The data shown in Figure 5E and Supplementary Figure S4A stem from ten single EV preparations (293T, Calu-3, Caco-2, HepG2, Huh7 cells with and w/o VSV-G) and EV transfer experiments using 17 different cell types as recipients in (technical) triplicates. The heat map is thus based on 170 single measurements. We added this information in the legend of these two figures in our revised manuscript.

5/ Figure 6B: I find it confusing that the authors changed the order of the bar graph in the left and right panels. I would recommend the author to be consistent between the two.

Reviewer 3 ‘comments’ on this point regarding our revised version (Oct 21) by stating that “…the authors modified Fig 6B to include additional baseline control for their experiment. It is an excellent improvement to their reported finding. Still, as initially commented, I would be happy that the authors keep the same order on the horizontal axis between mi.-BART1 and miR-BHRF-1-2 pannel.” Following this very thoughtful and considerate suggestion we adjusted the order of the results in the revised Figure 6B. The two panels show the identical order of miRNAs tested now.

6/ Figure S7B: report the data in "number of EVs / cells" instead of ul of EVs.

We fixed this issue and provide a revised version of Supplementary Figure S7B.
